# Diversity of Trends of Viremia and T-Cell Markers in Experimental Acute Feline Immunodeficiency Virus Infection

**DOI:** 10.1371/journal.pone.0056135

**Published:** 2013-02-07

**Authors:** Sylvain Roche, Hanane El Garch, Sylvie Brunet, Hervé Poulet, Jean Iwaz, René Ecochard, Philippe Vanhems

**Affiliations:** 1 Service de Biostatistique, Hospices Civils de Lyon, Lyon, France; 2 Université de Lyon, Lyon, France; 3 Université Lyon 1, Villeurbanne, France; 4 Equipe Biotatistique-Santé, Centre National de la Recherche Scientifique-Unité Mixte de Recherche 5558, Villeurbanne, France; 5 Discovery Research, Merial S.A.S., Lyon, France; 6 Service d’Hygiène, Epidémiologie et Prévention, Hospices Civils de Lyon, Lyon, France; 7 Equipe Epidémiologie et Santé Publique, Centre National de la Recherche Scientifique-Unité Mixte de Recherche 5558, Villeurbanne, France; Temple University School of Medicine, United States of America

## Abstract

**Objective:**

The early events of human immunodeficiency virus infection seem critical for progression toward disease and antiretroviral therapy initiation. We wanted to clarify some still unknown prognostic relationships between inoculum size and changes in various immunological and virological markers. Feline immunodeficiency virus infection could be a helpful model.

**Methods:**

Viremia and T-cell markers (number of CD4, CD8, CD8β^low^CD62L^neg^ T-cells, CD4/CD8 ratio, and percentage of CD8β^low^CD62L^neg^ cells among CD8 T-cells) were measured over 12 weeks in 102 cats infected with different feline immunodeficiency virus strains and doses. Viremia and T-cell markers trajectory groups were determined and the dose-response relationships between inoculum titres and trajectory groups investigated.

**Results:**

Cats given the same inoculum showed different patterns of changes in viremia and T-cell markers. A statistically significant positive dose-response relationship was observed between inoculum titre and i) viremia trajectory-groups (r = 0.80, p<0.01), ii) CD8β^low^CD62L^neg^ cell-fraction trajectory-groups (r = 0.56, p<0.01). Significant correlations were also found between viremia and the CD4/CD8 ratio and between seven out of ten T-cell markers.

**Conclusions:**

In cats, the infectious dose determines early kinetics of viremia and initial CD8+ T-cell activation. An expansion of the CD8β^low^CD62L^neg^ T-cells might be an early predictor of progression toward disease. The same might be expected in humans but needs confirmation.

## Introduction

Past observations have repeatedly suggested that early events in acute human immunodeficiency virus (HIV) infection may be critical for the outcome of infection and the progression toward disease [1]–[3] but the present extensive use of highly-active antiretroviral therapy might have decreased the impact of acute HIV infection on the outcome. Yet, the pathogenesis of the early phase still needs to be further explored to better understand its impact on the outcome of infection [4]–[6]. In addition, the relationships between the viral load and the changes in various immunological and virological markers are still poorly known [7], [8].

Because data on acute HIV infection are difficult to collect, interesting results might come from animal models such as the simian or the feline immunodeficiency virus (SIV or FIV) infection [9], [10]. Although the routes of infection, the primary receptors, and the genomic structures of FIV and HIV are different, the immunopathogenesis of FIV infection is similar to that of HIV [11]. Precisely, FIV infection results in an overall decrease of CD4+ T cells, an activation of CD4+CD25+ regulatory T cells, a dysregulation of cytokines, and a general immune hyperactivation leading to AIDS [12].

In various models, the inoculum size affected the outcome of infection [13]. In one of the few studies of the impact of the HIV viral load in the donor on the outcome of infection in the recipient partner, a correlation was found between donor and recipient viral loads [14]. However, that correlation reflected rather the fitness than the amount of virus and the study did not investigate the outcome of infection. A previous study has shown that various pathogenesis indicators were positively correlated with the FIV inoculum titre [15]. This issue is relevant for HIV because the semen HIV viral load is variable.

Because the inoculum titre may play a major role on the initial viral burst and the subsequent immune responses and changes in lymphocyte populations [16], a longitudinal study was carried out to monitor the diversity of changes in viremia and various T-cell markers during experimental acute FIV infection according to the inoculum titre. Besides, because usual statistical approaches failed to show patterns of changes over time, the heterogeneity of trends was dealt with using a group-based trajectory modelling. The relevance of the FIV model to HIV infection is discussed.

## Materials and Methods

### Ethics Statement

All animal experiments were conducted in accordance with the European Community regulations (Directive 2003/65/EC of the European Parliament and of the Council of 22 July 2003 amending Council Directive 86/609/EEC on the approximation of laws, regulations and administrative provisions of the Member States regarding the protection of animals used for experimental and other scientific purposes) and all procedures were supervised and approved by Merial Ethical Committee. This Committee, officially accredited by the French Ministry of Research and Education, examines the ethical issues relative to experiments on animals and checks the implementation of the European Community regulations.

### Study Setting and Data Collection

The dataset originated from longitudinal experiments on cats and pooled from various FIV vaccine calibration protocols. The dataset was also used in another study by Ribba et al.” (Ribba et al. (2012) Computational and Mathematical Methods in Medicine. Article ID 342602, 9 pages. doi:10.1155/2012/342602).” All animals were specific-pathogen free kittens purchased from Charles River laboratories (Lentilly, France). All the cats used in the study had the same genetic background.

At baseline, 102 cats (49 males and 53 females; mean age: 22.8 weeks, SD: 7.7, range: 13–36.5) were randomized to different inoculum groups within parentage and sex strata and infected for the first time with a single 1 mL of viral suspension (*Petaluma clade A, Glasgow-8 clade A, or EVA clade B*) via intra-muscular route (lumbar area). The virus stocks derived from plasma of infected cats. Inoculums were primary isolates from plasma amplified by a single passage on Mya-1 cells. No negative controls were included.

Preliminary in vivo experiments have shown that the three strains had comparable virulence in terms of viremia and impact on lymphocyte sub-populations. Virus dilutions ranged from 1/90,000 to 1/3 and the titres, calculated on Mya-1 T cells and expressed in log_10_/mL of Cell Culture Infectious Dose 50% (CCID50), ranged from 0.25 to 4.2 (Table 1). Three groups of cats were obtained with the quartiles of the distribution of the inoculum titre among the 102 cats: the first (1.25 log_10_/mL of CCID50) and the third quartile (3.6 log_10_/mL of CCID50) led to the following groups: ]0;1.25] (39 cats), ]1.25;3.6[(36 cats), and [3.6;4.2] (27 cats). Cats with titre 3.6 were included in the third group to ensure a sufficient number of cats in this group (Table 1). The cats were clinically examined weekly and bled under general anaesthesia.

**Table 1:** Number of infected cats according to the virus strain and the inoculum titre.

	Titre (log_10_/mL of CCID50)										
	0.25	0.7	1.25	1.3	1.7	2.6	2.7	3.2	3.6	3.7	4.2
EVA clade B		5		5		11					
Glasgow-8 clade A					4			4	5	5	
Petaluma clade A	6	6	22				12		12		5

Viremia and T-cell markers were measured at baseline (time 0, before virus inoculation) and at the ends of weeks 1, 3, 4, 6, 9, and 12. As carried out earlier [17], viremia was measured by qRT-PCR (quantitative reverse transcription polymerase chain reaction) after checking the absence of mismatch between the sequence of the amplified region of the virus and the sequence used to design the PCR primers and probe. Also, the lymphocyte populations were characterized by flow cytometry using the same set of monoclonal antibodies [17].

**Figure 1:**
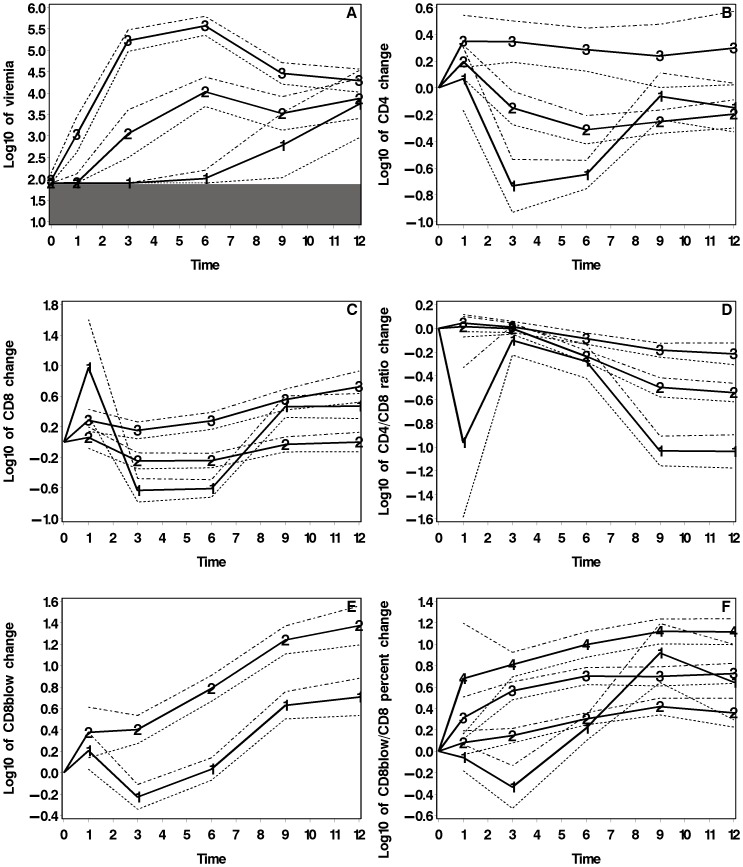
Trajectory-groups. Trajectory-groups (with 95% confidence intervals) for viremia and changes in T-cell markers over time since contamination (in weeks). Viremia is expressed in log_10_ of viral RNA copies per mL of plasma. The hatched area indicates the area below the virus detection threshold (1.9). Each T cell marker is expressed as the predicted value of log_10_(*X*_w_/*X*_0_) where *X*_0_ is the value at baseline and *X*_w_ the value at week *w* (1 to 12).

Viremia was expressed as log_10_ of viral RNA copies per mL of plasma. The detection threshold was 80 copies per mL (1.9 on the log_10_-scales in Figure 1). Cats with undetectable viremia (i.e., 11 animals with a titre below 1.3) were kept in the present statistical analysis.

The T-cell markers included the CD4 and CD8 cell counts, the CD4/CD8 ratio, the CD8β^low^CD62L^neg^ cell count, and the percentage of CD8β^low^CD62L^neg^ among CD8 cells (herein noted percent CD8β^low^/CD8). First, the CD4, CD8, and CD8β^low^CD62L^neg^ cell counts were expressed in thousands of cells per mL of blood then these counts, the CD4/CD8 ratio, and the percent CD8β^low^/CD8 were transformed into changes as follows: log_10_ of the value at the end of a given week minus log_10_ of the value at baseline. This allowed underlining the changes along time and the overall trends.

### Statistical Analysis

The plots of changes in viremia and T-cell markers values versus time in cats having had different inoculums showed a high heterogeneity between animals. Usual statistical approaches such as mixed models have shown the mean trends in viremia and T-cell markers [18] but were unable to depict various patterns over time because they assume that a single set of parameters can depict changes over time. However, when a single trajectory shape cannot fit the sample or the distribution of the outcome is unknown, a group-based trajectory modelling can estimate sets of parameters that define shapes of trajectories and probabilities of trajectory-group membership. Thus, individual differences in trajectories can be summarized by a small number of polynomial functions of time, each of which corresponding to a trajectory-group; i.e., individuals that follow approximately the same variation. Trajectory-groups can be thought of as fictional categories approximating the unknown population distribution.

Models with various numbers of trajectory-groups were fitted for each variable. Thus, the finally retained number was determined statistically. The optimal number of trajectory-groups was determined according to the highest probability of “correct model” given by a formula that uses a Bayesian Information Criterion (BIC) [19]–[21], except the optimal number of CD4/CD8 ratio trajectory-groups that was determined by an empirical method (the BIC for that ratio was mathematically inadequate).

The trajectory-groups with their confidence intervals were graphed for viremia and each T-cell marker and the percentages of the whole cat population in each trajectory calculated. The presence of a detection threshold for viremia led us to analyse viremia values as left-censored.

The dose-response relationships between inoculum titre and trajectory-groups were assessed by Mantel-Haenszel trend tests. The relationships between inoculum titre, trajectory-groups of viremia, and trajectory-groups of T-cell markers were assessed using polychoric correlations.

Trajectory-group models were sought using SAS PROC TRAJ [22]. All statistical analyses used SAS software, version 9.1.3. All tests were two-tailed and p<0.05 was considered for statistical significance.

## Results

### Determination of the Optimal Number of Trajectory-groups

Table 2 shows the BIC values and the derived probabilities of “correct model” for each variable and various numbers of trajectory-groups. The highest probability indicates the optimal number of trajectory-groups to consider: 2 for CD8β^low^CD62L^neg^, 3 for viremia, CD4 and CD8, and 4 for the percent CD8β^low^/CD8.

**Table 2:** Criteria for the choice of the optimal number of trajectory-groups.

Parameters and number of trajectories	Bayesian Information Criterion	Probability of “correct model”
Viremia		
2 trajectories	−580.24	0.00
3 trajectories	−560.06	0.99
CD4		
2 trajectories	−77.41	0.14
3 trajectories	−75.98	0.60
4 trajectories	−76.86	0.25
CD8		
2 trajectories	−118.74	0.00
3 trajectories	−110.70	0.99
CD8β^low^CD62L^neg^		
2 trajectories	−155.70	0.99
3 trajectories	−162.62	0.00
Percent CD8β^low^/CD8		
2 trajectories	−58.09	0.00
3 trajectories	−54.82	0.12
4 trajectories	−52.81	0.88

### Descriptions of the Trajectory-groups

Table 3 shows the optimal number of trajectory-groups per variable, describes the typical changes during the first week and the rest of follow-up, and gives the percentages of the whole cat population in each trajectory-group.

**Table 3:** Aspect of the trajectories of viremia and changes in T-cell markers.

Parameters and trajectories	Trajectory aspect		Proportion of cats
	Week 1	Weeks 2 to 12	
Viremia			
Trajectory 1	Flat	Protracted and moderate increase	19.1%
Trajectory 2	Flat	Moderate increase	35.3%
Trajectory 3	Steep increase	Steep increase then stability	45.6%
CD4			
Trajectory 1	Slight increase	Very steep decrease then sharp increase	32.0%
Trajectory 2	Moderate increase	Slight decline	56.4%
Trajectory 3	Steep increase	Flat	11.6%
CD8			
Trajectory 1	Very steep increase	Very steep decrease then sharp increase	28.1%
Trajectory 2	Slight increase	Slight decrease	43.7%
Trajectory 3	Moderate increase	Slight decrease then slight increase	28.3%
CD4/CD8 ratio			
Trajectory 1	Very steep decrease	Very steep increase then steep decrease	6.4%
Trajectory 2	Slight increase	Moderate decrease	42.6%
Trajectory 3	Flat	Slight decrease	51.0%
CD8β^low^CD62L^neg^			
Trajectory 1	Moderate increase	Moderate decrease then moderate increase	57.5%
Trajectory 2	Steep increase	Moderate increase	42.5%
Percent CD8β^low^/CD8			
Trajectory 1	Slight decrease	Slight decrease then steep increase	14.5%
Trajectory 2	Slight increase	Slight increase	43.0%
Trajectory 3	Moderate increase	Slight increase	28.1%
Trajectory 4	Very steep increase	Slight increase	14.5%

Figure 1 shows six panels, one per variable. Trajectory-group names and orders are independent between panels because these trajectory-groups are not permanent categories that include the same cats. For example, cats that follow Trajectory 3 for viremia may be different from those that follow Trajectory 3 for the change in CD4 cell count.

Panel A of Figure 1 shows the three trajectory-groups for viremia. Trajectory 3 showed a steep increase after FIV infection. It represented 45.6% of the cat population. Trajectories 1 and 2 (19.1% and 35.3% of cat population, respectively) could not show raises in viremia before one- or three-week delay, respectively, because these periods were concealed by the use of value 1.9 as detection threshold. The overlapping of the confidence intervals late during follow-up and for relatively short periods indicates that the three trajectories were well-differentiated. Significantly distinct between week 3 and week 8, the trajectories for viremia ended at approximately the same level at week 12.

The three trajectories of the change in CD4 cell counts showed different rates of increase during the first week post-infection (Figure 1, Panel B). Afterwards, only Trajectory 3 (11.6% of cats) remained stable while Trajectories 1 and 2 (32.0% and 56.4% of cats) showed transient declines. These trajectories were significantly distinct between weeks 3 and 6, but only Trajectories 2 and 3 remained significantly distinct thereafter. This suggests an association between the changes during the first week and those seen during the following weeks.

Concerning the change in CD8 cell counts, Trajectory 2 represented 43.7% of the cats. Trajectories 2 and 3 remained clearly distinct throughout the follow-up. Trajectory 1 showed a very steep decrease after week 1 followed by a sharp increase. Trajectories 1 and 3 were not significantly distinct after week 9 (Figure 1, Panel C).

Concerning the change in the CD4/CD8 ratio, all three trajectories decreased at different rates after week 3. Trajectory 1 gathered only 6.4% of the cat population. The two other trajectories involved similar percentages of cats and decreased moderately. The three trajectories were significantly distinct after week 7 (Figure 1, Panel D).

Concerning the change in CD8β^low^CD62L^neg^ cell counts, the two trajectories showed increases starting from week 3 but at very different levels (see confidence intervals, Figure 1, Panel E).

Concerning the change in the percent CD8β^low^/CD8, three of four trajectories were perfectly distinct and progressed parallelly. Thus, there might be an association between the variation during the first week post-infection and that seen afterwards. Trajectory 1 gathered 14.5% of cat population and showed a slight decrease then a steep increase (Figure 1, Panel F).

In this approach, no predictor for trajectory-group membership was previously specified. More specifically, age and sex were not used to construct the trajectory groups of viremia or T-cell markers. These variables, as shown in Table 4, show little differences related to age or sex between trajectory groups.

**Table 4:** Sex and age of cats by trajectory group for viremia and changes in T-cell markers.

Parameters and trajectories	Number of cats	Sex	Age in weeks (mean±SD)
Viremia			
Trajectory 1	20	12 females (60%)/8 males (40%)	24.2±7.3
Trajectory 2	36	19 females (52.78%)/17 males (47.22%)	26.2±6.7
Trajectory 3	46	22 females (47.83%)/24 males (52.17%)	19.6±7.5
CD4			
Trajectory 1	26	16 females (61.54%)/10 males (38.46%)	19.0±6.2
Trajectory 2	67	33 females (49.25%)/34 males (50.75%)	24.1±8.0
Trajectory 3	9	4 females (44.44%)/5 males (55.56%)	24.1±6.2
CD8			
Trajectory 1	24	13 females (54.17%)/11 males (45.83%)	16.8±3.8
Trajectory 2	57	28 females (49.12%)/29 males (50.88%)	25.6±7.6
Trajectory 3	21	12 females (57.14%)/9 males (42.86%)	22.4±7.6
CD4/CD8 ratio			
Trajectory 1	5	3 females (60.00%)/2 males (40.00%)	17.3±9.1
Trajectory 2	32	19 females (59.38%)/13 males (40.63%)	20.3±7.9
Trajectory 3	65	31 females (47.69%)/34 males (52.31%)	24.5±7.1
CD8β^low^CD62L^neg^			
Trajectory 1	69	34 females (49.28%)/35 males (50.72%)	24.0±7.4
Trajectory 2	33	19 females (57.58%)/14 males (42.42%)	20.4±8.0
Percent CD8β^low^/CD8			
Trajectory 1	12	6 females (50.00%)/6 males (50.00%)	22.3±5.3
Trajectory 2	57	30 females (52.63%)/27 males (47.37%)	25.6±6.9
Trajectory 3	21	9 females (42.86%)/12 males (57.14%)	16.3±6.8
Trajectory 4	12	8 females (66.67%)/4 males (33.33%)	21.8±8.5

### Mean Trend and Heterogeneity of Trends

The trajectory-groups show that a mean trend does not describe well biological phenomena such as viremia and changes in T-cell markers. For example, for CD4 (Figure 1, Panel B), all cats had an increase during the first week after infection. Afterwards, Groups 1 and 2 cats had a temporary decrease while Group 3 cats kept stable values.

A joint reading of Table 5 and Figure 1 illustrates the diversity of trends regarding a given marker among a trajectory-group of another marker. For example, in the low-viremia group (Group 1, Panel A), the percent CD8β^low^/CD8 could show either a constant slight increase (Group 2, Panel F) or an irregular progress (Group 1, Panel F). However, in the early-increase viremia groups (Group 2 or 3, Panel A), the percent CD8β^low^/CD8 only increased thought at different levels (Groups 2, 3 and 4, Panel F). Cats whose CD4 did not decrease (Group 3, Panel B) had rather low viremias (Group 1, Panel A) and also low percent CD8β^low^/CD8 (Groups 1 and 2, Panel F).

**Table 5:** Cross-tabulation of trajectory-groups according to viremia and changes in T-cell markers during the 12-week period after FIV inoculation.

Parameters andtrajectories	Viremia			CD4			CD8		
	1	2	3	1	2	3	1	2	3
CD4									
1	5^a^	8	13						
2	10	25	32						
3	5	3	1						
CD8									
1	5	7	12	19	5	0			
2	8	21	28	7	49	1			
3	7	8	6	0	13	8			
Percent CD8β^low^/CD8									
1	7	5	0	0	6	6	0	5	7
2	13	27	17	16	39	2	12	37	8
3	0	0	21	10	11	0	10	7	4
4	0	4	8	0	11	1	2	8	2

a The content of each cell is the number of cats among the 102 cats of the study.

### Relationships between Variations of Viremia and T-cell Markers

As shown in Table 6, there were no significant correlations between variations in viremia and changes in CD4, CD8, and CD8β^low^CD62L^neg^ cell counts. A significant negative correlation was observed between viremia and the change in the CD4/CD8 ratio, as well as a significant positive correlation between viremia and the change in the percent of CD8β^low^/CD8. There were significant positive correlations between the following changes: i) CD4 and CD8 counts, ii) CD4 and CD8β^low^CD62L^neg^ counts, iii) CD8 and CD8β^low^CD62L^neg^ counts, iv) CD8β^low^CD62L^neg^ count and the percent CD8β^low^/CD8. There were significant negative correlations between the following changes: i) CD8 count and the percent CD8β^low^/CD8; ii) the CD4/CD8 ratio and CD8β^low^CD62L^neg^ cell count; iii) the CD4/CD8 ratio and the percent CD8β^low^/CD8.

**Table 6:** Polychoric correlation coefficients between groups of inoculum and trajectory-groups of viremia and changes in T-cell markers during the 12-week period after virus inoculation.

Parameters	Inoculum	Viremia	CD4	CD8	CD4/CD8 ratio	CD8β^low^ CD62L^neg^	Percent CD8β^low/^CD8
Inoculum	1						
Viremia	0.80†	1					
CD4	−0.19	−0.23	1				
CD8	−0.16	−0.18	0.88†	1			
CD4/CD8 ratio	−0.02	−0.32*	0.08	−0.20	1		
CD8β^low^CD62L^neg^	0.14	0.19	0.54†	0.51†	−0.63†	1	
Percent CD8β^low^/CD8	0.56†	0.70†	−0.31	−0.30*	−0.34†	0.49†	1

Significance:

† p<0.01,

* p<0.05,

Polychoric correlations are not correlations between quantitative variables but correlations between ordinal variables; here, the trajectory-groups. For example, CD4 and CD8 are viewed as ordinal variables, each with three categories. Using the cross-tabulation of CD8 by CD4 given in Table 4, a polychoric correlation of 0.88 means that cats that belong to group 3 for CD4 have a higher probability of belonging also to group 3 for CD8 than to the two other groups. Conversely, 0.08 means that cats that belong to group 3 for CD4 have a low probability of belonging to group 3 for CD4/CD8 ratio and could belong to any of the three groups for the CD4/CD8 ratio.

### Dose-response Relationship between Inoculums and Trajectory-groups

A statistically significant dose-response relationship was found between inoculum titre and viremia as well as between inoculum titre and the change in the percent CD8β^low^/CD8 (p<0.0001). Table 6 shows that there was a significant positive correlation between viremia and inoculum titre (r = 0.80, p<0.01) and another between the change in the percent CD8β^low^/CD8 and inoculum titre (r = 0.56, p<0.01). The higher was the inoculum titre, the steeper was the increase of viremia and the change in the percent CD8β^low^/CD8. The p values for the trend test between inoculum titre and changes in the counts of CD4, CD8, and CD8β^low^CD62L^neg^ cells, and CD4/CD8 ratio were 0.19, 0.24, 0.3 and 0.84, respectively.

## Discussion

The follow-up of cohorts of HIV infected individuals suggested that high viral load and CD4 cell loss at seroconversion are early markers of disease progression [23]. The impact of the initial burst of HIV replication on the outcome of infection and the progression toward disease remain poorly understood, mainly because data collection on HIV patients during acute infection is difficult. Animal models may therefore help better characterize that early phase. Indeed, FIV and HIV infections have a similar immunopathogenesis and comparable viral load kinetics; thus, FIV is a natural model of HIV [6]. Here, we analyze the early virological and immunological data from a cohort of cats after experimental FIV infection.

To our knowledge, this is one of the first applications of the trajectory-group approach in the field of FIV or HIV infection. Thus, it should be stressed beforehand “that individuals do not actually belong to a trajectory-group, that the number of trajectory-groups in a sample is not immutable, and that individuals do not follow the group-level trajectory in lock step” [24]. In other words, the trajectories that model marker changes along time should not be considered as a classification but the major patterns of change. This approach was of great help in disentangling the observed heterogeneity and offering debatable biological trends.

In the present study, the groups of viremia over three months were positively correlated with the groups of FIV inoculum titre. This titre affected the magnitude and the timing of viremia peak, two comparable characteristics between FIV and HIV infections. All viremia trajectories ended with similar values at week 12 post-infection suggesting that the viremia set-point values were comparable whatever the inoculum. In HIV, the viremia set-point has been already proposed as a predictive marker of disease progression [25]–[28]. The convergence of the trajectories toward the same viremia set-point may be explained by the homogeneity of our FIV model in terms of animal genetic background and FIV strain virulence.

The inoculum viral load determined the expansion of CD8β^low^CD62L^neg^ cells, the circulating effector T cells with antiviral activity. This expansion is a hallmark of HIV and FIV infections [29], [30], [31]. Those activated CD8 T cells have a strong antiviral activity [17], [31] but are prone to apoptosis [17], [32]. The maintenance of a high percentage of activated CD8 cells is a consequence of a hyperactivation of the immune system associated with the viral burden in FIV infected cats. The sustained expansion of the CD8β^low^CD62L^neg^ cell population is specific to FIV and is not observed in other feline retroviral infections such as infection by the feline leukaemia virus. At the peak of viremia, the expansion of CD8β^low^CD62L^neg^ cells was the only parameter correlated with the infectious titre of the inoculum. Interestingly, whereas the trajectories for viremia became indistinguishable at week 12, the trajectories of the percent CD8β^low^/CD8 cells showed different levels of CD8-cell activation. Over the twelve-week follow-up period, viremia and changes in percent CD8β^low^/CD8 cells were positively correlated. Importantly, viremia was correlated with the change of percent CD8β^low^/CD8 but not with the changes in CD8β^low^CD62L^neg^ or CD8 cell counts. In this study, we measured the rate of activation of the CD8 cell population but did not characterize the phenotypes or differentiation statuses of all CD8 subpopulations. In early infection, the proportion of some of those subpopulations, like naïve CD8+ T cells, may be predictive of HIV disease progression [33]. The balance between activated CD8 cells and other CD8-cell subsets such as central memory T cells might be more critical than the count of activated CD8 cells.

The correlations between trajectory-groups in the present study showed that viremia and changes in percentage of CD8β^low^CD62L^neg^ cells were associated with a lower CD4/CD8 ratio, another feature of FIV-induced immune dysregulation. We have also found a negative correlation (though non-significant) between changes in viremia and changes in the CD4+ T-cell counts. A negative correlation between viral load in plasma and CD4+ T-cell counts has been observed in early HIV infection [34]. In the simian HIV model, a correlation was found between the peak viral load and the extent of CD4 T-cell depletion in acute infection [35].

One asset of this study is the experimental design with randomized groups of cats, which adjusts for differences between cats. Besides, the present results are consistent with observations in a cohort of HIV patients [34]. These indicate that the early CD8 T-cell activation (as supported by the percent CD8β^low^/CD8) was correlated with the initial viremia and that the immunologic activation set-point was established in the early phase of infection. In HIV patients, the progress of CD8 T-cell activation and viremia were associated with decline of CD4 cell counts. Interestingly, CD8-cell activation was also positively correlated with the number of symptoms observed during the acute phase of infection [34]. Although clinical signs in acute FIV infection are difficult to observe (transient hyperthermia, lymphadenopathy, dehydration diarrhoea, gingivitis), there is a correlation between their intensity and early viremia (data not shown), which is consistent with another observation [15]. A positive relationship between clinical signs in acute infection, viral load, and T-cell activation has also been observed in acute HIV infection [34]. In addition, clinical observations of HIV infected patients have shown relationships between the intensity of the acute phase of infection or the delay to appearance of acute signs and the progression toward disease [2], [36]. Overall, those independent observations suggest that the intensity of the initial viral burst and the extent of early T-cell activation might have an impact on the progression toward disease.

Our study has shown that the inoculum titre determined the initial kinetics of viremia as well as the expansion of the CD8β^low^CD62L^neg^ cell population. FIV-specific T-cell responses were also related to the inoculum titre (data not shown). On week 12, most cats had similar viremias, but displayed different lymphocyte population profiles. FIV strain virulence may play an important role; however, here, the cats were infected with strains of similar virulence to avoid a bias in the analysis of the impact of the inoculum titre. Since our FIV strains have not been cloned, it might be argued that the composition of the quasispecies administered depends on the inoculum titre. In fact, the virus stocks have been amplified in cats; it is therefore unlikely that the quasispecies contain a subdominant variant with high virulence.

The infection dose is critical in models that test HIV or FIV vaccine candidates [16], [37]. Our study focused on the impact of the inoculum titre on the kinetics of viremia and some T-cell populations, but the infection dose may also be critical in determining the magnitude of the immune response [13].

### Conclusions

The virological and immunological group-specific trends in more than a hundred FIV-infected cats showed that the inoculum titre determined the early kinetics of viremia and initial activation of CD8 cells; thus, the outcome of infection. Despite similar viremia three months after infection, the animals displayed group-specific trends and three different levels of CD8-cell activation. The early expansion of the CD8β^low^ CD62L^neg^ T-cell population might be an early predictive marker of disease progression in FIV infected cats. Because several features of FIV infection parallel those reported in HIV infection, the latter expansion might also happen in humans. Thus, investigations focussed on this twelve-week period might provide an appropriate confirmation and be of real help in detecting early HIV infection and starting early therapy.

## References

[1] Schacker TW, Hugues JP, Shea T, Coombs RW, Corey L (1998) Biological and virologic characteristics of primary HIV infection. Ann Intern Med 128: 613–620. 9537934 10.7326/0003-4819-128-8-199804150-00001

[2] Vanhems P, Lambert J, Cooper DA, Perrin L, Carr A, et al (1998) Severity and prognosis of acute human immunodeficiency virus type 1 illness: a dose-response relationship. Clin Infect Dis 26: 323–329. 9502449 10.1086/516289

[3] Kassutto S, Rosenberg ES (2004) Primary HIV Type 1 Infection. Clin Infect Dis 38: 1447–1453. 15156484 10.1086/420745

[4] Pope M, Haase AT (2003) Transmission, acute HIV-1 infection and the quest for strategies to prevent infection. Nat Med 9: 847–852. 12835704 10.1038/nm0703-847

[5] Pilcher CD, Eron JJ Jr, Galvin S, Gay C, Cohen MS (2004) Acute HIV revisited: new opportunities for treatment and prevention. J Clin Invest 113: 937–945. 15057296 10.1172/JCI21540 PMC379335

[6] Sparger E (2006) FIV as a model of HIV: An Overview. In: Friedman H, Spector S, Bendinelli M, editors. In vivo models of HIV disease and control. Infectious Agents and Pathogenesis series. New York: Springer. 189–207.

[7] Sprague WS, TerWee JA, VandeWoude S (2010) Temporal association of large granular lymphocytosis, neutropenia, proviral load, and FasL mRNA in cats with acute feline immunodeficiency virus infection. Vet Immunol Immunopathol 134: 115–121. 19896217 10.1016/j.vetimm.2009.10.016 PMC2821998

[8] Rychert J, Strick D, Bazner S, Robinson J, Rosenberg E (2010) Detection of HIV gp120 in Plasma During Early HIV Infection Is Associated with Increased Proinflammatory and Immunoregulatory Cytokines. AIDS Res Hum Retroviruses 26: 1139–1145. 20722464 10.1089/aid.2009.0290 PMC2982714

[9] Tompkins MB, Nelson PD, English RV, Novotney C (1991) Early events in the immunopathogenesis of feline retrovirus infections. J Am Vet Med Assoc 199: 1311–1315. 1666073

[10] Elder JH, Lin YC, Fink E, Grant CK (2010) Feline immunodeficiency virus (FIV) as a model for study of lentivirus infections: parallels with HIV. Curr HIV Res 8: 73–80. 20210782 10.2174/157016210790416389 PMC2853889

[11] Burkhard MJ, Dean GA (2003) Transmission and immunopathogenesis of FIV in cats as a model for HIV. Curr HIV Res 1: 15–29. 15043209 10.2174/1570162033352101

[12] Tompkins MB, Tompkins WA (2008) Lentivirus-induced immune dysregulation. Vet Immunol Immunopathol 123: 45–55. 18289702 10.1016/j.vetimm.2008.01.011 PMC2410212

[13] Asabe S, Wieland SF, Chattopadhyay PK, Roederer M, Engle RE, et al (2009) The size of the viral inoculum contributes to the outcome of hepatitis B virus infection. J Virol 83: 9652–9662. 19625407 10.1128/JVI.00867-09 PMC2748002

[14] Hecht FM, Hartogensis W, Bragg L, Bachetti P, Atchison R, et al (2010) HIV RNA level in early infection is predicted by viral load in the transmission source. AIDS 24: 941–945. 20168202 10.1097/QAD.0b013e328337b12e PMC2887742

[15] Hokanson RM, TerWee J, Choi IS, Coates J, Dean H, et al (2000) Dose response studies of acute feline immunodeficiency virus PPR strain infection in cats. Vet Microbiol 76: 311–327. 11000529 10.1016/s0378-1135(00)00263-7

[16] Hosie MJ, Beatty JA (2007) Vaccine protection against feline immunodeficiency virus: setting the challenge. Aust Vet J 85: 5–12. 17300445 10.1111/j.1751-0813.2006.00071.x

[17] Paillot R, Richard S, Bloas F, Piras F, Poulet H, et al (2005) Toward a detailed characterization of feline immunodeficiency virus-specific T cell immune responses and mediated immune disorders. Vet Immunol Immunopathol 106: 1–14. 15910988 10.1016/j.vetimm.2004.12.023

[18] Thiébaut R, Jacqmin-Gadda H, Chêne G, Leport C, Commenges D (2002) Bivariate linear mixed models using SAS proc MIXED. Comput Methods Programs Biomed 69: 249–256. 12204452 10.1016/s0169-2607(02)00017-2

[19] Nagin D (1999) Analyzing Developmental Trajectories: A Semi-parametric, Group-based Approach. Psychol Methods 4: 139–177. 10.1037/1082-989x.6.1.18 11285809

[20] Schwarz G (1978) Estimating dimensions of a model. Ann Stat 6: 461–464.

[21] Kass RE, Wasserman LA (1995) Reference Bayesian test for nested hypotheses and its relationship to the Schwarz criterion. J Am Stat Assoc 90: 928–934.

[22] Jones B, Nagin D, Roeder KA (2001) SAS Procedure Based on Mixture Models for Estimating Developmental Trajectories. Sociol Methods Res 29: 374–393.

[23] Buchacz K, Hu DJ, Vanichseni S, Mock PA, Chaowanachan T, et al (2004) Early markers of HIV-1 disease progression in a prospective cohort of seroconverters in Bangkok, Thailand: implications for vaccine trials. J Acquir Immune Defic Syndr 36: 853–860. 15213570 10.1097/00126334-200407010-00013

[24] Nagin D, Tremblay R (2005) Developmental trajectory groups: fact or a useful statistical fiction. Criminology 43: 873–904.

[25] Ho DD (1996) Viral counts count in HIV infection. Science 272: 1124–1125. 8638155 10.1126/science.272.5265.1124

[26] Mellors JW, Rinaldo CR Jr, Gupta P, White RM, Todd JA, et al (1996) Prognosis in HIV-1 infection predicted by the quantity of virus in plasma. Science 272: 1167–1170. 8638160 10.1126/science.272.5265.1167

[27] Bajaria SH, Webb G, Cloyd M, Kirschner D (2002) Dynamics of naive and memory CD4+ T lymphocytes in HIV-1 disease progression. J Acquir Immune Defic Syndr 30: 41–58. 12048362 10.1097/00042560-200205010-00006

[28] Geskus RB, Prins M, Hubert JB, Miedema F, Berkhout B, et al (2007) The HIV RNA setpoint theory revisited. Retrovirology 4: 65. 17888148 10.1186/1742-4690-4-65 PMC2206052

[29] Willett BJ, Hosie MJ, Callanan JJ, Neil JC, Jarrett O (1993) Infection with feline immunodeficiency virus is followed by the rapid expansion of a CD8+ lymphocyte subset. Immunology 78: 1–6. 8094707 PMC1421779

[30] Papagno L, Spina CA, Marchant A, Salio M, Rufer N, et al (2004) Immune activation and CD8+ T-cell differentiation towards senescence in HIV-1 infection. PLoS Biology 2: 173–185. 10.1371/journal.pbio.0020020 PMC340937 14966528

[31] Gebhard DH, Dow JL, Childers TA, Alvelo JI, Tompkins MB, et al (1999) Progressive expansion of an L-selectin-negative CD8 cell with anti-feline immunodeficiency virus (FIV) suppressor function in the circulation of FIV-infected cats. J Infect Dis 180: 1503–1513. 10515809 10.1086/315089

[32] Appay V, Papagno L, Spina CA, Hansasuta P, King A, et al (2002) Dynamics of T cell responses in HIV infection. J Immunol 168: 3660–3666. 11907132 10.4049/jimmunol.168.7.3660

[33] Ganesan A, Chattopadhyay PK, Brodie TM, Qin J, Gu W, et al (2010) Infectious Disease Clinical Research Program HIV Working Group. Immunologic and virologic events in early HIV infection predict subsequent rate of progression. J Infect Dis 201: 272–284. 20001854 10.1086/649430 PMC2939466

[34] Deeks SG, Kitchen CM, Liu L, Guo H, Gascon R, et al (2004) Immune activation set point during early HIV infection predicts subsequent CD4+ T-cell changes independent of viral load. Blood 104: 942–947. 15117761 10.1182/blood-2003-09-3333

[35] Davenport MP, Zhang L, Shiver JW, Casmiro DR, Ribeiro RM, et al (2006) Influence of peak viral load on the extent of CD4+ T-cell depletion in simian HIV infection. J Acquir Immune Defic Syndr 41: 259–265. 16540926 10.1097/01.qai.0000199232.31340.d3

[36] Vanhems P, Hirschel B, Phillips AN, Cooper DA, Vizzard J, et al (2000) Incubation time of acute human immunodeficiency virus (HIV) infection and duration of acute HIV infection are independent prognostic factors of progression to AIDS. J Infect Dis 182: 334–337. 10882619 10.1086/315687

[37] Regoes RR, Longini IM, Feinberg MB, Staprans SI (2005) Preclinical assessment of HIV vaccines and microbicides by repeated low-dose virus challenges. PLoS Med 2: e249. 16018721 10.1371/journal.pmed.0020249 PMC1176242

